# DETERMINANTS OF SLEEP HEALTH IN THE CONTEXT OF THE COVID-19 PANDEMIC AMONG OLDER CHINESE IMMIGRANTS IN THE U.S

**DOI:** 10.1093/geroni/igae098.4159

**Published:** 2024-12-31

**Authors:** Stephanie Lyn Marquez, Wendi Da, Fengyan Tang, Yanping Jiang

**Affiliations:** Rutgers, The State University of New Jersey, New Brunswick, New Jersey, United States; Rutgers, the State University of New Jersey, New Brunswick, New Jersey, United States; University of Pittsburgh, Wexford, Pennsylvania, United States; Rutgers, The State University of New Jersey, New Brunswick, New Jersey, United States

## Abstract

Good sleep is crucial for healthy aging. While recent studies have examined psychosocial factors related to sleep health, their impact on older Asian immigrants is not well understood. Notably, few studies have examined how psychosocial factors and immigration factors may intensify the COVID-19 pandemic’s effect on sleep among this population. This study investigated how these factors, assessed prior to the pandemic, affected sleep health during COVID-19 among older Chinese immigrants. Data were drawn from the Population Study of Chinese Elderly (PINE), focusing on participants who completed the Pittsburgh Sleep Quality Index and Women’s Health Initiative Insomnia Rating Scale in wave 6 (2021-2023) and at least one prior wave between wave 4 (2017-2019) and wave 5 (2019-2021). A mixed-effects linear spline model analyzed predictors across five domains: individual characteristics (age, sex, marital status, education, income, health literacy, chronic conditions), psychological distress (depressive symptoms, anxiety, perceived loneliness), immigration factors (years living in the U.S., acculturation), interpersonal factors (social support, social strain, filial piety receipt and expectations), and perceived neighborhood environment (disorder and cohesion), all adjusted in the context of COVID-19. Results were stratified by residential environment (living in Chinatown versus living outside of Chinatown). The findings revealed that the pandemic worsened sleep health, with depressive symptoms being significantly related to a faster decline in sleep quality and faster increase in insomnia, regardless of residential environment. These findings suggest that the pandemic intensified the influence of psychosocial and immigration factors on sleep health among older Chinese immigrants, highlighting the need for targeted interventions.

